# Reducing dietary intake of added sugars could affect the nutritional adequacy of vitamin A in adolescents: the Costa Rica case

**DOI:** 10.1186/s12889-023-17243-w

**Published:** 2023-12-14

**Authors:** Rafael Monge-Rojas, Luis A. Barboza, Rulamán Vargas-Quesada

**Affiliations:** 1 Nutrition and Health Unit, Researcher, Costa Rican Institute for Research and Education On Nutrition and Health (INCIENSA), 4-2250 Tres Ríos, Cartago, Costa Rica; 2 Center for Pure and Applied Mathematics (CIMPA), Researcher, Department of Mathematics, Universidad de Costa Rica, San José, 2060 Costa Rica

**Keywords:** Added sugar, Vitamin A, Sugar fortification, Adequate intake, Adolescents, Costa Rica

## Abstract

**Background:**

In countries where sugar fortification with vitamin A is mandatory, strategies to reduce the prevalence of overweight/obesity in adolescents that involve lowering added sugar intake could lead to vitamin A inadequate intakes, since vitamin A-fortified sugar for home consumption contributes to a high proportion of this vitamin intake in the adolescent diet.

**Methods:**

The study employed a hierarchical linear model to perform a mediation analysis on a cross-sectional sample of adolescents (13–18 years old) in the province of San José, Costa Rica.

**Results:**

Lowering the total energy intake derived from added sugars to less than 10% significantly increases the prevalence of vitamin A inadequate intake in adolescents by 12.1% (from 29.6% to 41.7%). This is explained by the mediation model in which, the reduced adequacy of vitamin A intake is mediated by a reduction in total energy intake derived from added sugars fortified with vitamin A.

**Conclusions:**

The vitamin A fortification of sugar for household consumption should be reassessed according to the current epidemiological profile in Costa Rica to promote strategies that reduce the prevalence of overweight/obesity in adolescents by lowering the consumption of added sugars without affecting vitamin A intake.

## Background

High intakes of added sugars are associated with obesity among adolescents [1–3]. This fact is relevant for Costa Rican public health because 21% and 9.8% of adolescents (13-19 years old) are overweight or obese, respectively [4].

Added sugars affect the interaction of neural and hormonal mechanisms, leading to reactivity towards the consumption of palatable foods and the ensuing cycle of overeating and increased excessive weight [5]. Considering this, the 2015–2020 Dietary Guidelines for Americans [6], the most recent version of these guidelines [7], and the 2022 Dietary Guidelines Based on Food Systems for Adolescent and Adult Population in Costa Rica [8], have recommended the percentage of daily total energy intake from added sugars (TEI-AS) be less than 10%. However, less than 10% of Costa Rican adolescents meet this recommendation and about 70% consume more than 15% TEI-AS [9].

In Costa Rica, 74.0% of the added sugars consumed by adolescents are provided at home. Nearly 30% of this total is contributed by *frescos*, a Costa Rican traditional homemade beverage made by blending pieces of fresh fruit or fruit juice, sugar, and water [9]. It is important to note that, in Costa Rica, the sugar for household consumption is fortified with 6–20 µg/g of vitamin A (vit-A) [10] to ensure that it contains, on average, 8–9 µg/g of vit-A [11].

However, considering the high prevalence of overweight/obesity among adolescents, the high consumption of added sugars, and the potentially significant amount of vit-A that added sugars could provide, a paradox arises for Costa Rican public health to reduce or prevent obesity during adolescence. Lowering the added sugar intake to less than 10% TEI could lead to a markedly inadequate vit-A intake in adolescents, especially since adolescents are poor consumers of animal food sources of vit-A and vit-A-rich fruits and vegetables [12].

**Fig. 1:**
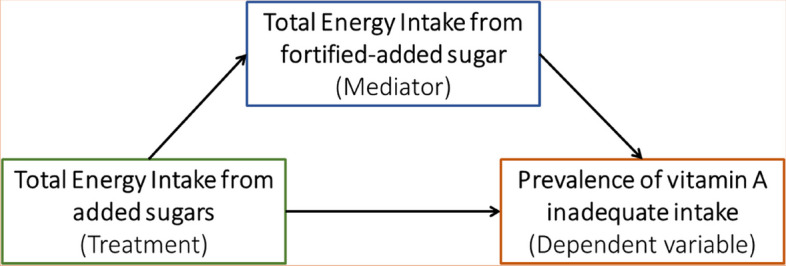
Conceptual model depicting the reduction of the adequacy of vitamin A intake due to the effect of lowering total energy intake from added sugars (TEI-AS), mediated by the reduction of total energy intake from fortified-added sugar (TEI-FAS)

The objective of this study was to perform, through a statistical mediation analysis, a projection of the effect of reducing the dietary intake of added sugars on the nutritional adequacy of vit-A in a sample of Costa Rican adolescents. As shown in the conceptual model (Fig. 1), this study hypothesizes that the inadequacy of vit-A increases when reducing the added sugar intake to less than 10% TEI, mediated by a reduction in the total energy intake from fortified sugar (TEI-FAS).

## Methods 

### Study population and setting

The data came from a cross-sectional sample of adolescents (13–18 years old; 7-11th graders) enrolled in 18 schools (10 urban and 8 rural) in the province of San José, Costa Rica, in 2017. Most Costa Rican adolescents (80%) are enrolled in school [13], and San José has the highest adolescent concentration (30%) in the country [14]. The study sample was estimated as previously described [9]. Students with signed informed assent and signed informed consent were selected to participate in the study. The final study sample was 818 adolescents aged 13 to 18 years. There was no sampling strategy of any kind regarding the determination of TEI from added sugars.

The study protocol was approved by the Bioethics Committee of the Costa Rican Institute for Research and Education on Nutrition and Health (INCIENSA) under number IC-2007–01.

### Sociodemographic variables

A paper-based questionnaire was used to collect data on sex, age, area of residence, parental education level, ownership of goods and access to services (e.g., computers, internet, router, cable television, and water heating for the whole house), and family structure. The information on educational level and ownership of goods and access to services was used to classify the socioeconomic status (SES) using the k-means procedure [15].

### Anthropometric assessment

Trained nutritionists measured height and weight following standardized procedures [16]. The body mass index (BMI) value for each participant was calculated from measured height and weight values using the standard equation: weight (kg)/height (m)^2^. Nutritional status was determined using the BMI Z-score for age, as recommended by World Health Organization [17]: < -2: underweight; ≥ -2 and <  + 1: normal weight; ≥  + 1 and <  + 2: overweight, and ≥  + 2: obesity. The categories of overweight and obesity were combined to provide a practical operationalization and interpretation of the weight status variable. Therefore, the nutritional status was operationalized as “overweight/obesity” vs. “non-overweight”.

### Dietary intake assessment

Dietary intake data were collected via 3-day food records completed by the participants in real-time and reviewed by nutritionists. Participants were asked to complete a 3-day food record (two weekdays and one weekend day). Data were collected during nine months of the school year (February-November), reflecting seasonal variations for Costa Rica: rainy season (May–November) and dry season (December–April).

Epi Info™ software, version 3.5.4 [18], was used to process data from the 3-day food records, and the food and nutrients database of the School of Nutrition of the University of Costa Rica was used with this tool. Data were reported as means ± standard deviations (SD) for continuous variables and frequencies (%) for categorical variables.

### Usual dietary intake

Multiple Source Method (MSM; https://msm.dife.de/tps/en), a web-based statistical modeling technique proposed by the European Prospective Investigation into Cancer and Nutrition (EPIC), was used to estimate usual energy and nutrient intakes [19]. The usual dietary intake is estimated with MSM by a 3 step-procedure: First, the probability of eating a specific food on a random day is estimated for each participant. Second, the usual amount of dietary intake on a consumption day is estimated using the inter- and intra-individual variance. Third, the resulting numbers from the first two steps are multiplied, to estimate the usual daily intake for each participant [20].

### Vitamin A assessment

Dietary intake of vit-A was estimated using the database of the School of Nutrition of the University of Costa Rica [21]. Dietary intake of vit-A was determined per person per day and included vit-A from animal and vegetable sources and foods fortified with this nutrient in Costa Rica (sugar for household consumption and commercially pasteurized cow milk).

The nutritional adequacy of vit-A intake was calculated using the Estimated Average Requirement (EAR) for each category of sex and age of adolescents, according to the guidelines of the National Academy of Medicine (formerly the Institute of Medicine) of the United States [22]. Nutrient adequacy was calculated using the cut-off point method [23], as shown in Eq. 1.

1 $$\%EAR=\left(\frac{Usual\;Dietary\;Intake\;of\;Vitamin\;A}{Vitamin\;A\;EAR}\right)\times100$$

The EAR cut-point method was used because all the assumptions the Institute of Medicine indicated for its use were met [24]. A vit-A percentage of EAR (%EAR) less than 100% was considered indicative of inadequate vit-A intake [24].

### Added sugars assessment

The dietary intake of added sugars was estimated as previously described [9]. Given that the World Health Organization (WHO) does not provide a specific recommendation for added sugars, the analyses used the recommendation of consuming < 10% TEI from added sugars (TEI-AS) established by the 2015–2020 edition of the Dietary Guidelines for Americans (DGA) as a basis for comparison [6].

### Statistical analyses

The statistical analyses were performed in two steps. First, an exploratory analysis was performed to examine the association between some of the study variables. Second, an analysis was conducted to quantify the mediational effect on vit-A intake due to a change in the intake of added sugars.

### Exploratory analysis 

**Fig. 2:**
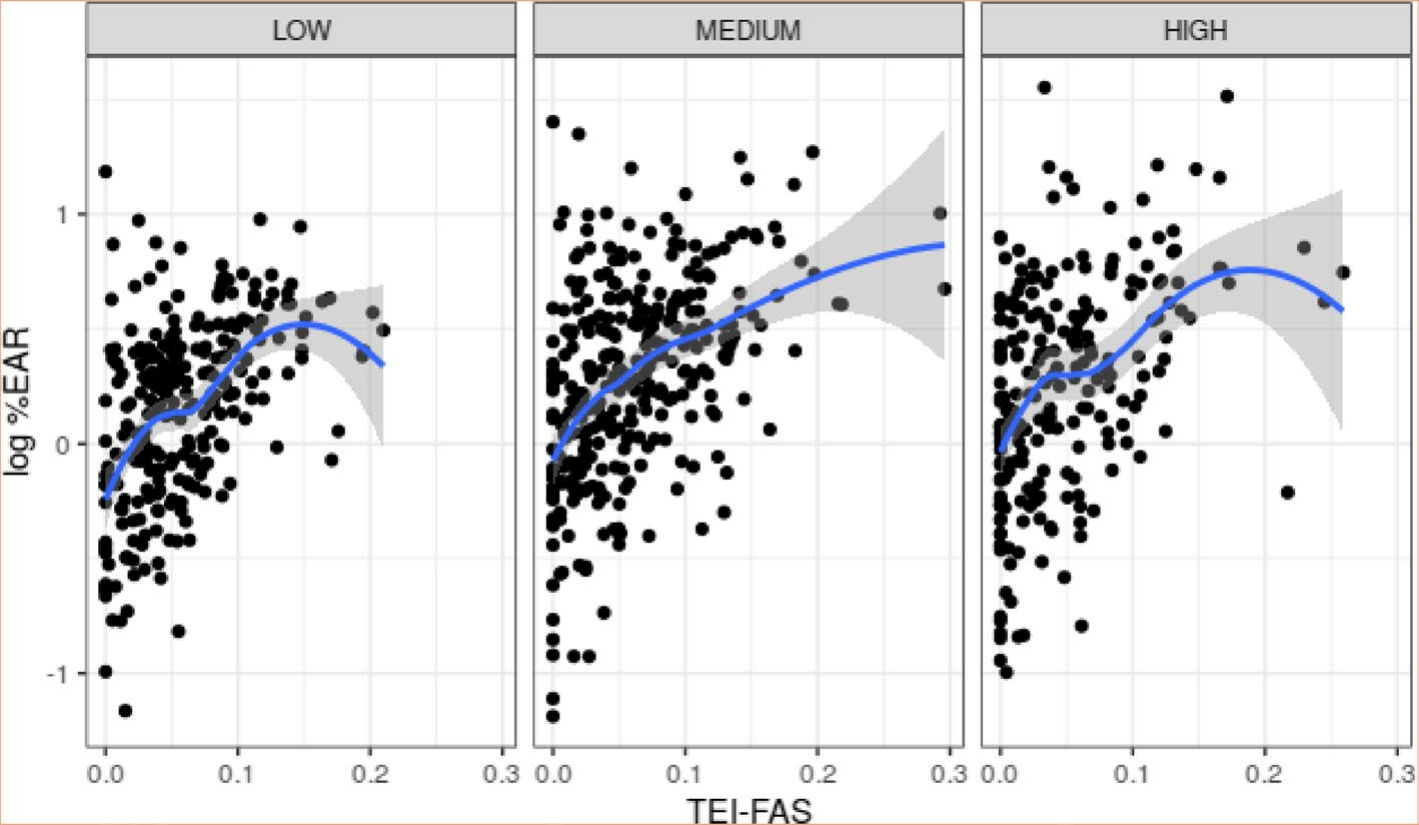
Association between the logarithm of %EAR (log %EAR) and TEI from fortified-added sugar (TEI-FAS) according to socioeconomic status

To determine the association between the variables included in the dataset, the correlation between quantitative variables was measured and the effect of the qualitative variables was analyzed using scatterplots and boxplots. In general terms, there is a positive association between log %EAR and TEI-FAS, regardless of socioeconomic status. The association between these variables was calculated using the Pearson correlation, which is 0.447 (*p* < 0.001). Moreover, for low and high socioeconomic statuses, a TEI-FAS level seems to exist such that there is a decrease in log %EAR for larger values of TEI-FAS. These details are shown in Fig. 2.

Furthermore, TEI-FAS appears to be slightly larger in rural versus urban areas. It is important to note that there is no substantial collinearity between TEI-FAS and TEI-AS, as evidenced by their Pearson correlation coefficient of approximately 0.316 (*p* < 0.001). This assures us that multicollinearity concerns are unlikely to affect the linear model we introduce in the following section.

### Statistical mediation model

The statistical model used in this study follows the ideas of Imai [25, 26] in the sense that a hierarchical linear model formed by output and mediator components can isolate the mediational effect on an output or dependent variable (in this case, %EAR) under a given level of a treatment variable (in this case, TEI-AS). To reduce the effect of confounding factors, the TEI-FAS variable was incorporated into the model as a mediator variable, based on the apparent linear relationship between TEI-FAS and TEI-AS.

Based on the previous exploratory analysis and taking prior evidence into account [9, 27], a linear output model with log %EAR as the dependent variable was defined, using sex, age, and socioeconomic status as covariates, TEI-FAS as the quadratic function, and TEI-AS as the treatment variable. On the other hand, the mediator model has TEI-FAS as the dependent variable, with socioeconomic status and residence area as covariates, and TEI-AS as the treatment variable. Therefore, the model can be written as follows:

2 $$\left\{\begin{array}{c}log \%EAR = {\alpha }_{0} + {\alpha }_{1}Sex + {\alpha }_{2}Age + {\alpha }_{3}{SES}_{Low} + {\alpha }_{4}{SES}_{Medium} + {\alpha }_{5}TEI -FAS + {\alpha }_{6}TEI - {FAS}^{2} + {\alpha }_{7}\chi TEI -AS, 1 + {\alpha }_{8}\chi TEI -AS, 2 + \varepsilon \\ TEI -FAS = {\beta }_{0} + {\beta }_{1}\chi TEI -AS, 1 + {\beta }_{2}\chi TEI -AS, 2 + {\beta }_{3}{SES}_{Low} + {\beta }_{4}{SES}_{Medium} + {\beta }_{5}{Area}_{Rural} + \eta \end{array}\right.$$

where:

- %EAR: nutrient adequacy, defined according to Eq. 1.
- *SES*: socioeconomic status.
- $TEI$-$FAS$: total energy intake from fortified-added sugar.
- $\chi TEI$-$AS:$ 0 if TEI-AS is less than 10%; 1 if TEI-AS is equal or greater than 10% and less than 20%, or 2 otherwise. The TEI-AS term is repeated because it is a categorical covariate with three levels, and then it is represented as two dummy variables in the linear model.
- *Area:* residence area (rural, urban).
- $\varepsilon$ and $\eta$ are non-correlated centered Gaussian errors.

The output and mediator models defined in Eq. 2 were fitted using the R package *mediator* [28]. This package offers a bootstrap-based approach [29] to determine the uncertainty of the average causal mediation effect (ACME) estimates, with bias correction [30]. The calculation of ACME estimates was performed with 1000 samples. Level 0 of TEI-AS (less than 10%) was considered as the baseline treatment status.

The fitting process on both models should be done individually, and maximum-likelihood criteria were used to perform that estimation [25]. Once both models were fitted, the mediation effect [31] was estimated through ACME [25, 26] as a way to quantify the potential outcome that results from a treatment change through the mediator variable. In this process, it was assumed that the treatment variable (TEI-AS) was randomized throughout the study. Moreover, to compute ACME effectively, it was assumed that the mediator variable was completely randomized under fixed values of the remaining covariates and the treatment variables. These hypotheses can be confirmed due to the sampling features of the study.

The uncertainty of ACME estimates was computed using Bootstrap. Bootstrap-based confidence intervals have been recommended in previous studies for the estimation of mediation effects [29]. To improve the precision of the ACME estimates, the bias-corrected version of those intervals was used in the calculations.

A complementary analysis was conducted to confirm the mediating effect of reducing TEI-AS on vit-A adequacy. This involved applying the reduction percentages of the %EAR of vit-A according to changes in TEI-AS (according to the < 10% TEI recommendation) to the original %EAR of vit-A for each individual.

## Results

The mean age of the study sample was 15.3 y ± 1.8, with 63.9% girls, 50.2% urban residents, 32.6% with overweight/obesity, and 32.2% and 39.7% adolescents of low and middle socioeconomic status respectively (Table 1).

**Table 1:** General and dietary intake characteristics of the Costa Rican adolescents sample

Characteristics	*n* = 818^a^
**Study population**	
Age (years)	15.3 ± 1.8
Girls (%)	63.9
Urban (%)	50.2
Overweight/obesity (%)	32.6
**Socioeconomic status (%)**	
Low	32.2
Middle	39.7
High	28.1
**Total energy intake (TEI, kcal)**	1940 ± 603
**Sugar intake**	
Total sugar (g/d)	115.4 ± 51.4
Total added sugars (g/d)	94.5 ± 48.3
Added sugar fortified with vitamin A (g/d)	27.3 ± 25.0
Added sugars unfortified (g/d)	67.3 ± 44.6
**TEI from added sugars (TEI-AS, %)**^**b**^	
TEI < 10%	9.5
10% ≤ TEI < 20%	46.7
TEI ≥ 20%	43.8
**Dietary intake of vitamin A (µg/d)**	
Total vitamin A (µg/d)	699 ± 455
Vitamin A from fortified-added sugar (µg/d)	218 ± 200
Vitamin A from other food sources (µg/d)	480 ± 402
**Adequacy of vitamin A intake (%)**^**c**^	
≥ EAR	70.4
80–99.9% EAR	13.9
< 80% EAR	15.7
**Fortified-added sugar intake per range of vitamin A intake adequacy (g/d)**	
≥ EAR	33.8 ± 26.0
80–99.9% EAR	15.8 ± 15.1
< 80% EAR	8.0 ± 8.2

*Abbreviations*: *TEI* total energy intake, *TEI-AS* TEI from added sugars, *EAR* Estimated Average Requirement

^a^Values are means ± SD or percentages unless otherwise indicated

^b^Proportion of adolescents according to their TEI-AS

^c^Proportion of adolescents according to their range of vitamin A intake adequacy

The mean added sugar intake was 94.5 g/d, of which 29% (27.3 g/d) came from vit-A fortified-added sugar. Only 9.5% of adolescents met the recommended dietary intake of added sugars (< 10% TEI), while 43.8% consumed more than 20% TEI-AS. The mean dietary intake of vit-A was 699 µg/d, of which 31% (218 µg/d) came from fortified-added sugar (FAS). About 70% of adolescents had an adequate intake of vit-A (EAR ≥ 100%) and around 30% had an inadequate intake. Adolescents with a vit-A adequate dietary intake (≥ EAR) consumed an average of 33.8 g/d of FAS; however, this consumption was 2–4 times lower in those with an inadequate intake. The output and mediator models defined in Eq. 2 were fitted separately using maximum-likelihood criteria, and the results are shown in Table 2.

**Table 2:** Estimated coefficients for the output and mediator models in a Costa Rican adolescents sample (*n* = 818)

Model	Term	Estimate	*p*-value^1^
**Output**	**Sex**		
	Girls	0.1265	< 0.0001
	**Age**	-0.0350	< 0.0001
	**Socioeconomic status**		
	Low	-0.1629	< 0.0001
	Medium	-0.0370	
	${\varvec{T}}{\varvec{E}}{\varvec{I}}$**-** ${\varvec{F}}{\varvec{A}}{\varvec{S}}$	6.3363	< 0.0001
	${\varvec{T}}{\varvec{E}}{\varvec{I}}$**-** ${{\varvec{F}}{\varvec{A}}{\varvec{S}}}^{2}$	-12.7405	< 0.0001
	${\varvec{\chi}}{\varvec{T}}{\varvec{E}}{\varvec{I}}$**-** ${\varvec{A}}{\varvec{S}}$		
	10% ≤ TEI < 20%	0.0584	0.1607
	TEI ≥ 20%	0.0082	
	**Intercept**	0.4198	< 0.0001
**Mediator**	**Residence area**		
	Rural	0.0086	0.0120
	**Socioeconomic status**		
	Low	0.0098	0.0290
	Medium	0.0109	
	${\varvec{\chi}}{\varvec{T}}{\varvec{E}}{\varvec{I}}$**-** ${\varvec{A}}{\varvec{S}}$		
	10% ≤ TEI < 20%	0.0312	< 0.0001
	TEI ≥ 20%	0.0513	
	**Intercept**	0.0083	0.1600

*Abbreviations*: *TEI* total energy intake, *TEI-AS* TEI from added sugars, *TEI-FAS* TEI from fortified-added sugar, $\chi TEI$*-* $AS$ TEI from added sugars in 3 categories (< 10%, 10% to < 20%, and ≥ 20%)

^1^*p*-value < 0.05 is statistically significant

Note that all the covariates in the output model are significant according to the F-test, except the TEI-AS indicator, which is significant only through the mediator model with the TEI-FAS dependent variable. Moreover, there is a significant quadratic relationship between TEI-FAS and log %EAR, suggesting that the growth rate of TEI-FAS is neither constant nor linear over log %EAR. Possibly because the relationship between both variables is not homogeneous between different individuals and the changes reflected in log %EAR do not depend exclusively on increases in TEI-FAS but also on other dietary factors associated with dietary intake of vit-A. The remaining covariates (SES and residence area) are also significant in the mediator model.

By using an exponential transformation on the ACME estimator, the reduction in the vit-A %EAR, due to a change in TEI-AS from between 10% and ≤ 20% to < 10%, was computed as 15.5% (CI 95% [12.2%, 19.2%]). When the change in TEI-AS was from ≥ 20% to < 10%, the respective reduction in vit-A %EAR was estimated as 23.4% (CI 95% [20.5%, 27.4%]). Both estimators show the causal mediational effect on the dependent variable using TEI-FAS as the mediator variable.

**Fig. 3:**
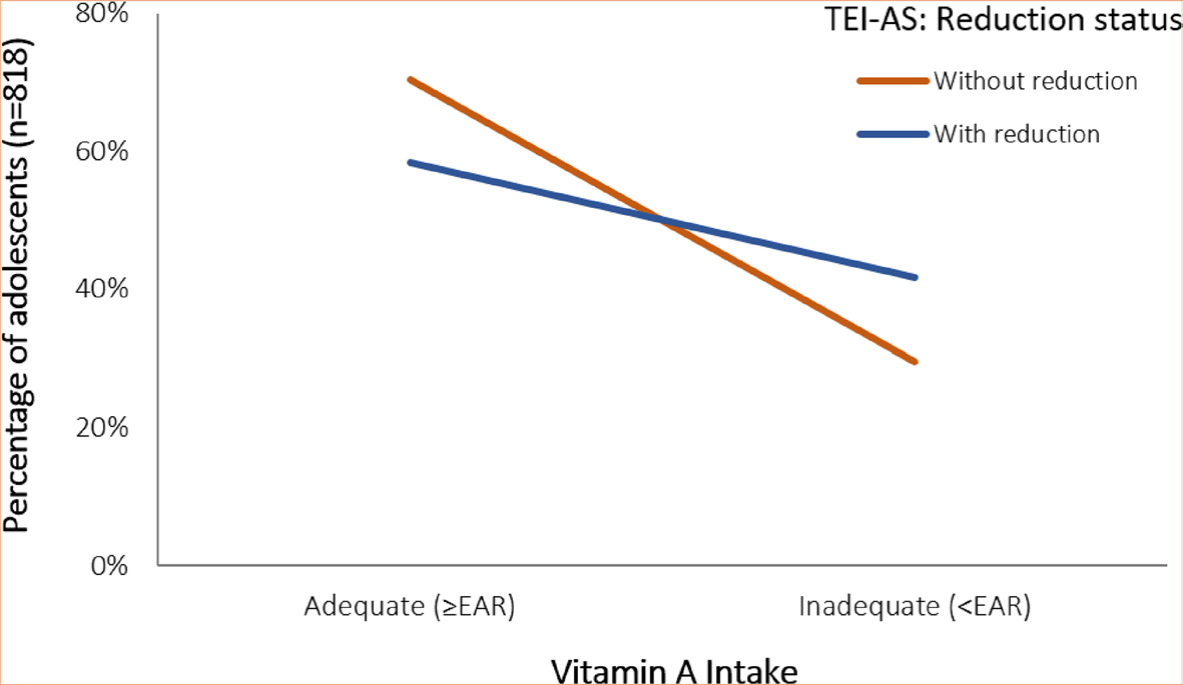
Potential increase in the prevalence of vitamin A inadequacy due to the effect of reducing total energy intake from added sugars (TEI-AS), mediated by total energy intake from fortified-added sugar (TEI-FAS)

The result of the mediating effect of reducing TEI-AS on vit-A adequacy (Fig. 3) showed that decreasing TEI-AS to less than 10% results in a significant increase in the prevalence of vit-A inadequacy among adolescents, by 12.1% (from 29.6% to 41.7%). Figure 3 also highlights the presence of an interaction between the average reduction of the TEI-AS variable and the indicator of vit-A intake adequacy. This relationship has been rigorously examined and statistically explained using the mediator model presented in Eq. 2. Moreover, the graphical association can be confirmed by the significant mediating effect observed in the output and mediator hierarchical models.

## Discussion

According to our hypothesis and the statistical mediator model developed for this study, reducing added sugar consumption significantly increases the proportion of Costa Rican adolescents with inadequate vit-A intake (from 29.6% to 41.7%), mediated by a reduction in TEI derived from added sugars fortified with vit-A.

Inadequate intake of vit-A has been identified as the leading cause of preventable childhood blindness and a major contributor to morbidity and mortality from infections in children and pregnant women in developing countries [32]. However, an inadequate dietary intake of vit-A could also have deleterious consequences for adolescent health. Vit-A is a crucial component of many important and diverse biological functions, including reproduction, embryological development, cellular differentiation, growth, immunity [33], and new biological functions for vit-A are continuously being discovered in new fields such as lipid metabolism, insulin response, cardiovascular disease, and obesity [34].

Vit-A intake does not correlate with serum retinol levels because 1) serum retinol levels are controlled homeostatically within a narrow range and do not change unless liver reserves are very low or high [35], 2) serum retinol levels increase during pubertal maturation in both boys and girls [36], and 3) due to oral contraceptive use in adolescent girls [37]. It has been pointed out that the effect of these factors on retinol levels does not necessarily reflect a good vit-A status, but merely that the body's retinol stores have been redistributed from the liver into circulation [37]. Meanwhile, an inadequate intake of vit-A for four months can lead to the depletion of retinol stores in the liver [38] and generate a deficiency in serum levels, as evidenced in adolescents in Brazil [39], China [40], the Philippines [41], Iran [42], and India [43]. Although it has been suggested that vit-A deficiency is manifested primarily as a subclinical disease in the Americas [44], it could generate deleterious effects on the health of adolescents through different pathways, such as those mentioned in the introduction [33, 34].

Although for the last five decades, the vit-A fortification of sugar for household consumption has indeed been very successful in reducing the prevalence of vit-A deficiency in Costa Rica [11], rethinking the vit-A fortification of sugar for household consumption would be advantageous for strategies aimed at preventing overweight/obesity and cardiometabolic diseases since a substantial reduction in vit-A intake in adolescents could be avoided. This study sheds new light on the dilemma that decisions taken to improve a public health problem in past decades could generate, given new challenges associated with the current epidemiological profile of a country.

The need to continue fortifying sugar for household consumption with vit-A in Costa Rica should be reconsidered, since this is the primary source of vit-A in the adolescent diet and its consumption significantly exceeds the recommended 10% TEI [9] and 21% and 9.8% of Costa Rican adolescents (13–19 years old) are overweight or obese, respectively [4]. This issue requires in-depth and critical analysis by public health stakeholders since one of the strategies promoted by the WHO to reduce the prevalence of obesity from an early age is to decrease the consumption of added sugars [45].

The epidemiological profile in Costa Rica has changed substantially since the fortification of sugar for household consumption with vit-A began. In the 1970s and 1980s, the high prevalence of malnutrition [46] and the control of vit-A deficiency achieved by fortifying sugar led to the fortification program being strongly promoted by the health authorities [47]. The health authorities have perpetuated the sugar fortification program, even when the prevalence of obesity in preschool and school children and adolescents has increased continuously since the 1990s [8] and the prevalence of vit-A deficiency (without correction for inflammatory markers: C-reactive protein and alpha-1-acid glycoprotein) was low in the last National Nutrition Survey carried out in 2008–2009 (preschool children: 2.8%; school children: 2.1%) [48]. Some authors have suggested that the prevalence of vit-A deficiency in Costa Rica could be somewhat overestimated due to the lack of adjustment for inflammatory markers [48]. In addition, there is no information on the prevalence of vit-A deficiency in Costa Rican adolescents.

The edible oils are an ideal matrix for fortification with vit-A which must be analyzed in Costa Rica and other contexts where vit-A fortification of sugar for household consumption is mandatory and the prevalence of overweight/obesity tends to increase in both children and adolescents. According to the International Vitamin A Consultative Group, the fortification method of edible oils is well-established, simple, and easy to implement at a low cost [49]. The fortification of edible oils would not promote their excessive intake; instead, it would foster the consumption of a healthy fatty acid profile. In the last decade, several countries worldwide have initiated national mandatory programs to fortify cooking oil with 15–30 µg/g of vit-A [50]. However, to date, there is no information available on the reduction of subclinical vit-A deficiency through fortification with edible oils [51].

Nevertheless, the use of sugar for household consumption or fortified edible oils as vehicles to improve vit-A intake could increase the risk of overweight/obesity in the population. Preferably, before promoting the consumption of high-caloric foods fortified with vitamin A, it is necessary to conduct nutritional interventions to reduce the consumption of these foods before adjusting fortification levels. Therefore, a public health strategy could promote the adequate intake of vit-A via the consumption of an amount of sugar or oils compatible with the prevention of overweight/obesity. In addition, it is crucial to promote the consumption of vit-A-rich fruits and vegetables, even though retinol conversion capacity of the provitamin-A carotenoids contained in these foods is also very low (between 3.6:1 and 28:1) [52].

### Study limitations and strengths

This study had several limitations including its cross-sectional design, which meant we could not examine causality. From a methodological point of view, mediation analysis (like any statistical methodology) has its limitations. For example, it relies on strong assumptions about the causal structure of the data [53], and there can be remaining unmeasured confounding variables in the analysis due to the simplification of the hierarchical models [26, 53]. However, the methodology used to develop the model is statistically robust and provides insights into the underlying causal mechanisms linking the output and treatment variables [54]. It also helps in identifying confounding variables by measuring the direct and indirect effects of the treatment variable on the outcome [25], and it can handle complex hierarchical relationships among the outcome, mediator, and treatment variables, including non-linear relationships [55].

## Conclusions

Lowering the consumption of added sugar to less than 10% TEI could significantly reduce the nutritional adequacy of vit-A intake and could increase the risk of vit-A deficiency in Costa Rican adolescents. The vit-A fortification of sugar for household consumption should be reassessed according to the prevailing epidemiological profile in Costa Rica to promote strategies that reduce the prevalence of overweight/obesity in adolescents by lowering the consumption of added sugars without affecting vit-A intake. The results of this study are relevant for Costa Rican public health, as well as for those countries where the fortification of sugar with vitamin A is mandatory and the prevalence of overweight/obesity in children and adolescents is increasing.

## Data Availability

The data underlying this article will be shared on reasonable request to the corresponding author.

## Abbreviations

ACME: Average Causal Mediation Effect
EAR: Estimated Average Requirement
SES: Socioeconomic Status
TEI: Total Energy Intake
TEI-AS: Total Energy Intake from Added Sugars
TEI-FAS: Total Energy Intake from Fortified-Added Sugar
Vit-A: Vitamin A
